# Reliability, factor structure, and criterion validity: testing the problematic social media use scale in Chinese college students

**DOI:** 10.7717/peerj.21138

**Published:** 2026-05-11

**Authors:** Manyun Li, Tieqiao Liu, Xuyi Wang, Yunfei Wang, Yuzhu Hao, Shubao Chen, Marc N. Potenza, Tania Moretta

**Affiliations:** 1 Department of Psychiatry, National Clinical Research Center for Mental Disorders and National Center for Mental Disorders, The Second Xiangya Hospital of Central South University, Changsha, China; 2 Department of Psychiatry, Hunan Second People’s Hospital (Hunan Brain Hospital), Changsha, China; 3 Department of Psychiatry & Clinical Psychology, The Seventh Affiliated Hospital, Sun Yat-sen University, Shenzhen, China; 4 Department of Psychiatry, Yale University School of Medicine, New Haven, CT, United States of America; 5 Child Study Center, Yale School of Medicine, New Haven, CT, United States of America; 6 Department of Neuroscience, Yale University, New Haven, CT, United States of America; 7 Wu Tsai Institute, Yale University, New Haven, CT, United States of America; 8 Connecticut Mental Health Center, New Haven, CT, United States of America; 9 Connecticut Council on Problem Gambling, Wethersfield, New Haven, CT, United States of America; 10 Department of Theoretical and Applied Sciences, eCampus University, Novedrate, Italy

**Keywords:** Chinese college students, Problematic use of social media, Validity, Addictive behaviors, Compulsive behaviors, Impulsive behaviors

## Abstract

**Background:**

Despite the increasing prevalence of problematic use of social media (PUSM) in the general population and in college students, there remains no fully agreed-upon clinical definition or assessment tool to identify and characterize PUSM. Caplan’s cognitive-behavioral model of generalized problematic use of the internet may provide a conceptual basis for understanding PUSM.

**Methods:**

The present study aimed to test the psychometric properties of Caplan’s model in the context of PUSM among Chinese college students. The Chinese version of the Problematic Social Media Use Scale (PSMUS, including five subscales: preference for online social interaction (POSI), mood regulation, cognitive preoccupation, compulsive use, and negative outcomes) was administered to Chinese college students.

**Results:**

Data from 788 students (mean age = 20.92 ± 2.74 years, 23.52% male) were analyzed. Confirmatory factor analysis supported the feasibility of Caplan’s model in the context of PUSM. Structural equation modeling revealed that POSI was a key factor which directly/indirectly (*via* mood-regulation-oriented PUSM) related to deficient self-regulation, which strongly related to negative social media outcomes (*β* = 0.85, *p* < .001), explaining 72% of their variance.

**Conclusion:**

The results support the adequate psychometric properties of the PSMUS in Chinese college students, thereby substantiating its utility in assessing PUSM in this population.

## Introduction

The use of social media continues to grow globally, with over half of the world’s population expected to be actively using by 2028 (Statista, 2025). The number of people using social media in China has reached 1.1 billion, including a vast majority of college students who engage with these platforms daily (China Internet Network Information Center, 2025). Social media can help build and maintain relationships, facilitate communication and share information across geographic boundaries (Kuss & Griffiths, 2017), but excessive use a may result in psychological distress, personal concerns and poor academic/occupational performance (Hosen et al., 2021; Hussain & Griffiths, 2021; Pirdehghan, Khezmeh & Panahi, 2021; Thomas et al., 2022; Xie et al., 2021). The maladaptive patterns of social media use have been described as problematic use of social media (PUSM) (Brand et al., 2025; Wu, Huang & Yang, 2024). Although there are no an uniformly agreed-upon features or formal diagnostic criteria for PUSM, a meta-analysis reported a pooled overall prevalence of 24% worldwide (Cheng et al., 2021), suggesting PUSM is a prevalent public health concern globally (Dell’Osso et al., 2021; Haddad et al., 2021; Office of the Surgeon General (OSG), 2023).

College students have been reported as a potential at-risk population for developing PUSM. Existing studies have shown significant global variations in the prevalence of PUSM among college students, ranging from 3.22% (the Netherlands) to 49.87% (Italy) (Bergaoui et al., 2024; Boer et al., 2020; Gopakumar et al., 2025; Hou et al., 2019; Li et al., 2025; Sampasa-Kanyinga & Lewis, 2015). Such variations may be attributed to the lack of an official definition, differences in assessment tools, cultural variations, and gender differences (Brand et al., 2020; Lee, Potenza & Bhang, 2025; Moretta et al., 2022), suggesting the importance of validating cross-culturally instruments such as the Problematic Social Media Use Scale (PSMUS). Additionally, the relationship between PUSM and psychological distress, insomnia and some compulsive behaviors has been previously reported (Brailovskaia & Margraf, 2017; Hou et al., 2019; Kolhar, Kazi & Alameen, 2021; Pirdehghan, Khezmeh & Panahi, 2021; Thomas et al., 2022). Over recent years, the mental health of college students has appeared to be declining (Halladay et al., 2024; Seehuus, Burt & Moeller, 2025). Especially in the setting of the COVID-19 pandemic and concurrent changes in digital technology use, the incidence of anxiety, depression, post-traumatic stress disorder (PTSD), and suicidal ideation has risen globally, including among Chinese students (LaMontagne et al., 2024; Luo, Zhong & Chiu, 2021; Seehuus, Burt & Moeller, 2025; Su et al., 2025). The motives for mood regulation and the interaction between PUSM and psychological distress may exacerbate these conditions (Soraci et al., 2025). Understanding and reliably measuring PUSM is therefore essential for developing targeted interventions.

PUSM should be conceptually distinguished from related constructs, such as PUI and smartphone overuse. PUI refers to a broad, multifaceted pattern of excessive and dysregulated internet use across various online activities (*e.g.*, gaming, information seeking, streaming) (Caplan, 2010; Davis, 2001), while smartphone overuse, in turn, centers on the excessive and compulsive use of the device itself, which often serves as the primary medium for accessing social media and other online applications (Soraci et al., 2025). PUSM is a specific manifestation focused on the maladaptive use of social networking platforms and their interactive features which can be understood as a specific subtype of problematic online behavior, frequently mediated by smartphone use, but defined by its core engagement with social interaction and content on dedicated platforms (Anish et al., 2024). Caplan’s model of generalized problematic use of the internet (PUI) (Caplan, 2003; Caplan, 2010) offers a theoretical framework for characterizing PUSM (Assunção & Matos, 2017; Marino et al., 2017; Moretta & Buodo, 2018). In this framework, PUSM would be characterized by some specific subdimensions, including preference for online social interaction (POSI), using social media for mood regulation purposes, cognitive preoccupation associated with the use of social media, compulsive use of social media, and negative outcomes (Caplan, 2010; Casale & Fioravanti, 2018; Marino et al., 2017; Moretta & Buodo, 2018; Musetti et al., 2022). The Generalized Problematic Internet Use Scale 2 (GPIUS2) (Caplan, 2010) was designed based on Caplan’s extension of a cognitive-behavioral theory (Davis, 2001), emphasizing the importance of POSI (Caplan, 2003) and considering the socio-cognitive model of PUI (Kim, LaRose & Peng, 2009; LaRose, Lin & Eastin, 2003). The GPIUS2 has been demonstrated good internal consistency and validity across versions (Assunção & Matos, 2017; Fioravanti, Primi & Casale, 2013; Laconi et al., 2018; Moretta & Buodo, 2018; Yoshimura et al., 2022; Zhang et al., 2024). However, its applicability to social media use among Chinese college students remains untested. Accordingly, the present study aims to build on and extend previous research about the reliability and validity of the Chinese version of the PSMUS in a sample of Chinese college students. Moreover, we studied whether the relationships among constructs proposed in Caplan’s cognitive-behavioral model were valid in the context of Chinese students. Specifically, we hypothesized the following in Chinese college students.

(H1) POSI would statistically predict the use of social media for mood regulation as well as deficient self-regulation;

(H2) Using social media for mood regulation would statistically predict deficient self-regulation of social media use, as well as mediate the relationship between POSI and poor self-regulation;

(H3) Deficient self-regulation would statistically predict negative outcomes of social media use, mediate the relationship between POSI and negative outcomes, and also mediate the relationship between mood regulation and negative outcomes.

## Materials & Methods

### Ethics statement

The study was conducted in accordance with the Declaration of Helsinki and was approved by the Institutional Review Board of the Second Xiangya Hospital of Central South University (NO. 2019-113). All subjects were duly informed about the study, and informed consent was obtained after the nature of the procedures had been fully explained.

### Participants

This anonymous web-based cross-sectional study was conducted using a QR code and website link delivery through specific social media platforms (WeChat, QQ Zone, and Douban) popular among Chinese college students. A 10-yuan mobile credit was provided as compensation upon submission of a valid response. Before answering questions, potential participants were asked to read the online informed consent and to click on the statement “I agree to the above consent form” or to log out. Those who agreed obtained access to the questionnaire, while others were shown an end page communicating appreciation. Participants needed to answer all questions before they were able to submit the questionnaire but could quit the questionnaire at any time. No participant quit before the end of the questionnaire. Inclusion criteria for manual review included using social media, being a college student, being 18 years or older. Exclusion criteria included uniform responses, incorrect screening answers (*e.g.*, National Day date), and overly rapid survey completion (<120s, based on a pilot study using the same procedure). A total of 788 college students (mean age = 20.92 ± 2.74 years, 23.52% male) were successfully recruited after a comprehensive manual review. Descriptive characteristics of the sample are reported in Table 1. The sample size met the recommended sample size for Structural Equation Modeling (SEM) (Violato & Hecker, 2007).

### Materials

Participants completed the Chinese version of the Problematic Social Media Use Scale (PSMUS) which was adapted from the GPIUS2 (Caplan, 2010) by replacing the word “internet” with “social media” where applicable. Permission was obtained from the original author before adoption. Moreover, the PSMUS was translated into Chinese by two of the authors and corrected from the professional, cultural, and linguistic perspectives. A translation back to English was done and reviewed by a separate bilingual psychiatrist to help ensure the accuracy of the translation, compared for differences and consistency between the Chinese and English versions, and repeatedly polished to align with Chinese national conditions and linguistic habits. The PSMUS includes 15 items rated on an 8-point Likert scale and five subscales: POSI, mood regulation, cognitive preoccupation, compulsive use, and negative outcomes. It is noteworthy that item 12 (“When offline, I have a hard time trying to resist the urge to go to social media”) has shown variability in factor loading across cultural adaptations, sometimes loading onto “POSI” or “cognitive preoccupation” in other populations (Cervin et al., 2022; Højgaard et al., 2017; Yoshimura et al., 2022). Higher PSMUS scores reflect more severe PUSM. The full list of items is reported in Table 2.

**Table 1:** Descriptive statistics of study variables (*N* = 788).

	Median	Interquartile range	Shapiro–Wilk statistic
Age	20	19–22	0.847^**^
Gender			
PSMUS	63	50–75	0.997
POSI	13	10–17	0.981^**^
Mood regulation	15	12–18	0.970^**^
Deficient self-regulation	42	17–31	0.985^**^
Cognitive preoccupation	12	8–16	0.972^**^
Compulsive use	12	9–12	0.978^**^
Negative outcomes	10	7–10	0.971^**^

**Notes.**

POSI stands for preference for online social interaction, ** represents *p* < .001.

**Table 2:** Internal consistency and factor loadings of the PSMUS items in the exploratory factor analysis.

	POSI	Mood regulation	Cognitive preoccupation	Compulsive use	Negative outcomes
Item 1	0.86				
Item 2	0.87				
Item 3	0.83				
Item 4		0.76			
Item 5		0.87			
Item 6		0.88			
Item 7			0.82		
Item 8			0.81		
Item 9			0.81		
Item 10				0.84	
Item 11				0.78	
Item 12	0.67			0.52	
Item 13					0.43
Item 14					0.83
Item 15					0.86
PV (%)	20.64	16.10	16.09	15.70	12.44
CV (%)	20.64	36.73	52.82	68.52	80.96

**Notes.**

POSI preference for online social interaction PV proportion variance CV cumulative variance

### Statistical analysis

All analyses were performed by IBM SPSS Statistics for Windows, version 26.0 (IBM Corp., Armonk, NY, USA) and Amos 24.0.0 version. Cronbach’s α was employed to assess the internal consistency of the scale, with values of 0.70 to 0.80 deemed acceptable, 0.80 to 0.90 deemed good, and above 0.90 deemed excellent.

Prior to exploratory factor analysis (EFA), data suitability was confirmed *via* Kaiser-Meyer-Olkin (KMO = 0.88) and Bartlett’s tests (*p* < .001). Principal component analysis using maximum likelihood estimation extracted five factors consistent with the GPIUS2 structure. Given prior evidence supporting both a five first-order and a four second-order factor structure (Caplan, 2010; Marino et al., 2017; Moretta & Buodo, 2018), Confirmatory Factor Analysis (CFA) was conducted for both models. Model fit was assessed by Chi-square (*χ*2) tests, Chi-square/degrees of freedom (*χ*2/d*f*, <5), standardized root mean square residual (SRMR, <0.08), root mean square error of approximation (RMSEA, 0.05 ∼ 0.08 acceptable, <0.05 good), comparative fit index (CFI, 0.95 ∼ 0.97 acceptable, >0.97 good), Tucker-Lewis index (TLI, 0.95 ∼ 0.97 acceptable, >0.97 good) (Schermelleh-Engel, Moosbrugger & Müller, 2003; Zhong et al., 2017). The initial model tested by the CFA was modified according to the Modification Indices (Table 3). Multigroup CFA was conducted to assess measurement invariance across sexes by a hierarchical approach. Finally, structural equation modeling (SEM) was used to evaluate the adequacy of Caplan’s second-order 4-factor model (Caplan, 2010).

**Table 3:** Confirmatory factor analysis and model modification steps of the Problematic Social Media Use Scale.

Indices	Initial model	First-order model	Second-order model
*χ* ^2^	504.46	323.06	350.27
*χ*^2^/d*f*	6.23	4.14	4.27
SRMR	0.05	0.04	0.04
RMSEA (90% CI)	0.082 (0.075, 0.088)	0.063 (0.056, 0.070)	0.064 (0.058, 0.072)
TLI	0.93	0.96	0.96
CFI	0.95	0.97	0.97

**Notes.**

Legend *χ*^2^ Chi-square df degrees of freedom SRMR standardized root mean square residual RMSEA root mean square error of approximation CI confidence interval CFI comparative fit index TLI Tucker-Lewis index

Initial model refers to the first fit of five factor model; First-order model refers to the model establishing the correlation of residual between items 9 and 12, and item 14 and 15 based on modification indices, which includes five factors with three items respectively, *i.e.*, preference for online social interaction (POSI), mood regulation, compulsive use, cognitive preoccupation, and negative outcomes; Second-order model includes four factor of POSI, mood regulation, deficient self-regulation (further including compulsive use and cognitive preoccupation), and negative outcomes.

## Results

The sociodemographic characteristics of the study sample are reported in Table 1.

### Item analysis

Independent sample *t*-test analysis showed statistically significant differences in each of the PSMUS items (all *p* < .001) between individuals with high levels of PUSM (scoring in the top 27%) and individuals with low levels of PUSM (scoring in the bottom 27%). The homogeneity test highlighted a significant positive correlation between the scores of each item and the total score (*r* = 0.52 − 0.79, all *p* < .001).

### EFA and reliability analysis

The EFA showed that the five-factor model was adequately supported, explaining 80.96% of the variance and exhibiting robust factor loadings (0.43–0.88; see Table 2). The five factors were designated as follows: preference for online social interaction (POSI), mood regulation (MR), cognitive preoccupation (CP), compulsive use (CU), and negative outcomes (NO). However, given that obsessive thoughts about social media may be more likely to be perceived as a loss of behavioral control rather than a preference for online interaction in Chinese cultural contexts, item 12 was loaded onto the “compulsive use” factor rather than the “POSI” factor even though it had factor loadings on both factors. The reliability analysis demonstrated good internal consistencies for both the PSMUS total score and its subdimensions (Cronbach’s α = 0.91, 0.79–0.90 for subscales). Cronbach’s α did not exhibit a statistically significant increase following the removal of any item.

### Confirmatory factor analysis and model revision process

The CFA was conducted according to the result of the EFA. The initial model exhibited satisfactory internal consistency but inadequate fit indices (*χ*2/d*f* = 6.23, RMSEA = 0.082, 90% CI [0.075–0.088], Table 3, column “initial model”). After establishing the correlation between items 9 and 12 and items 14 and 15 based on modification indices, the model showed a good fit (*χ*2/d*f* = 4.14, RMSEA = 0.063, 90% CI [0.056–0.070], Table 3 column “First-order model”).

**Figure 1:**
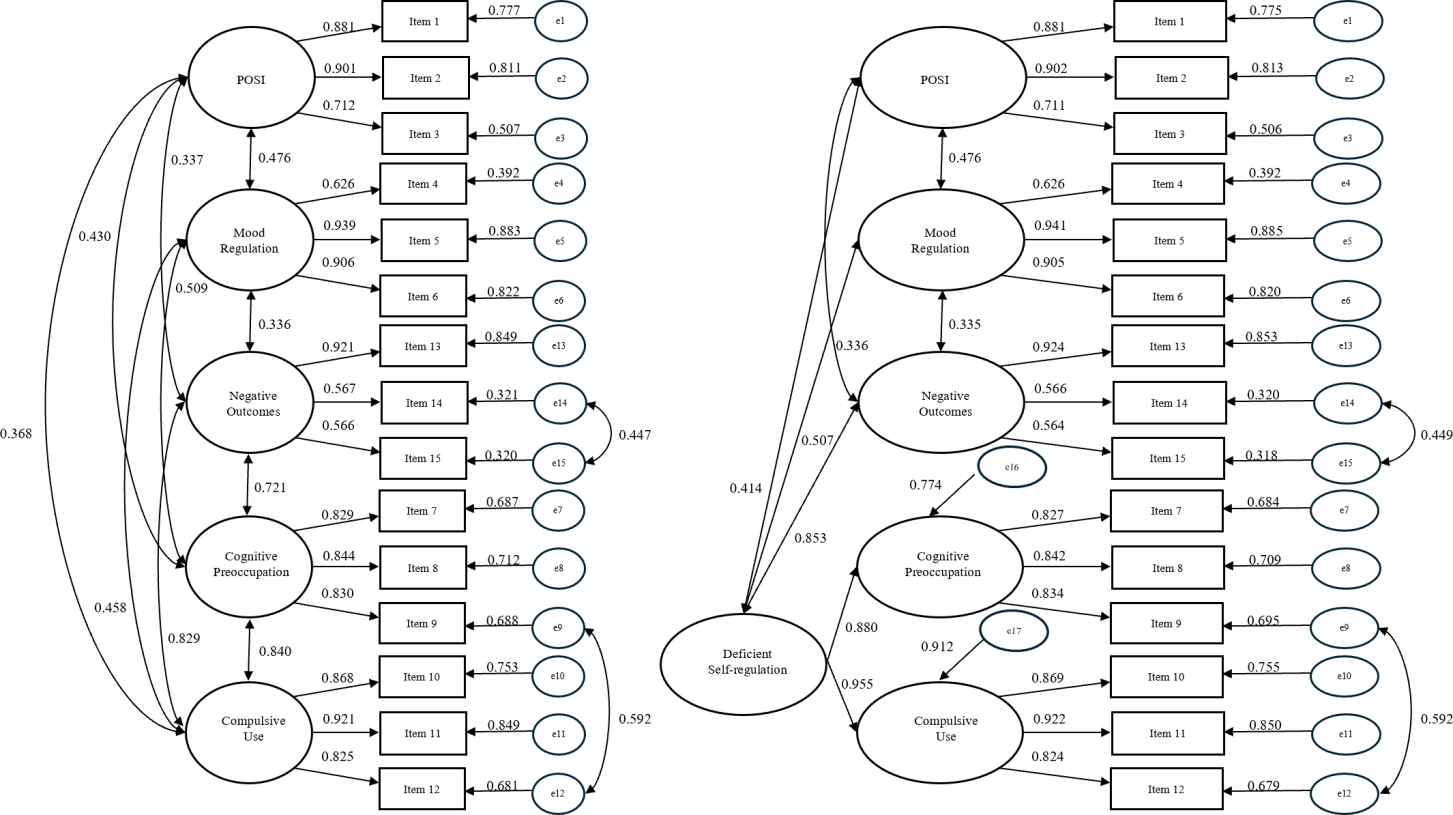
The first-order final model (left) and the second-order model (right) standardized path coefficient estimation results of PSMUS. POSI stands for preference for online social interaction.

In accordance with the high correlation between cognitive preoccupation and compulsive use factors and the original second-order 4-factor model of the GPIUS2, a second-order CFA was conducted. Results showed a good model fit (see Table 3 column “Second-order model”). The factor loadings ranged from 0.56 to 0.99 (*p* < .001) and the correlations between factors ranged from 0.34 to 0.85 (*p* < .001). The standardized estimates are shown in Fig. 1.

### Convergent validity, composite reliability, and discrimination validity

Pertinent indicators of convergent validity and composite reliability are reported in Table 4. The standard estimates ranged from 0.57 to 0.94 (*p* < .001), indicating a favorable interpretation of the items of each dimension. Average variance extracted (AVE) values were > 0.5 for each dimension, indicating favorable convergent validity. Finally, construct reliability values were over 0.7, supporting good composite reliability. The correlation coefficients of each dimension were less than the square root of AVE, indicating favorable discriminative validity of the scale (Table 5).

### Measurement invariance

The results of measurement invariance analysis are presented in Table 6. All models showed good fit (RMSEAs ≤ 0.03), and no significant differences across sexes were observed (ΔRMSEA < 0.015). These results indicated that the five-factor structure of the PSMUS exhibited good invariance with respect to sex in the unconstrained, measurement and structural models.

**Table 4:** Convergent validity and combination reliability of the Problematic Social Media Use Scale.

Dimension	Variable	Std. estimates	SMC	CR	AVE
POSI	Item 1	0.88^**^	0.78	0.87	0.70
	Item 2	0.90^**^	0.81		
	Item 3	0.71^**^	0.51		
Mood regulation	Item 4	0.63^**^	0.39	0.87	0.70
	Item 5	0.94^**^	0.88		
	Item 6	0.91^**^	0.82		
Cognitive preoccupation	Item 7	0.83^**^	0.69	0.88	0.71
	Item 8	0.84^**^	0.71		
	Item 9	0.83^**^	0.69		
Compulsive use	Item 10	0.87^**^	0.75	0.88	0.76
	Item 11	0.92^**^	0.85		
	Item 12	0.83^**^	0.68		
Negative outcomes	Item 13	0.92^**^	0.85	0.74	0.50
	Item 14	0.57^**^	0.32		
	Item 15	0.57^**^	0.32		
Deficient Self-regulation	CP	0.89^**^	0.786	0.94	0.88
	CU	0.99^**^	0.979		

**Notes.**

** Stands for *p* < .001.

POSI preference for online social interaction CU compulsive use CP cognitive preoccupation SMC squared multiple correlations CR construct reliability AVE average variance extracted

**Table 5:** Correlation coefficient, average variance extracted (AVE) and square root of AVE of the Problematic Social Media Use Scale model.

Dimension	POSI	Mood regulation	Cognitive preoccupation	Compulsive use	Negative outcomes
POSI	0.70				
Mood regulation	0.48^**^	0.70			
Cognitive preoccupation	0.43^**^	0.51^**^	0.71		
Compulsive use	0.38^**^	0.47^**^	0.84^**^	0.76	
Negative outcomes	0.34^**^	0.34^**^	0.75^**^	0.86^**^	0.50
Square root of AVE	0.84	0.84	0.85	0.87	0.70

**Notes.**

AVE average variance extracted POSI preference for online social interaction

** Stands for *p* < .001.

**Table 6:** Fit indices for measurement invariance tests on problematic social media use scale (PSMUS) across gender.

Model	*χ* ^2^	TLI	CFI	RMSEA (90% CI)	SRMR	ΔTLI	ΔCFI	ΔRMSEA
Unconstrained	630.91	0.969	0.976	0.033 (0.029, 0.036)	0.038			
Measurement weight	644.58	0.972	0.977	0.031 (0.028, 0.034)	0.038	−0.003	−0.001	−0.002
Measurement intercepts	660.06	0.976	0.978	0.029 (0.026, 0.032)	0.038	−0.004	−0.001	−0.002
Structural weights	660.44	0.976	0.978	0.029 (0.026, 0.031)	0.038	0	0	0.000
Structural covariance	660.44	0.978	0.978	0.027 (0.025, 0.030)	0.038	−0.002	0	−0.002
Structural residuals	674.68	0.978	0.978	0.027 (0.024, 0.030)	0.038	−0.001	0	0.000
Measurement residuals	708.46	0.981	0.978	0.026 (0.023, 0.028)	0.038	−0.002	0	−0.001

**Notes.**

*χ*^2^ Chi-square SRMR standardized root mean square residual RMSEA root mean square error of approximation CFI comparative fit index TLI Tucker-Lewis index CI confidence interval CFI comparative fit index TLI Tucker-Lewis index

### Structural Equation Model

**Figure 2:**
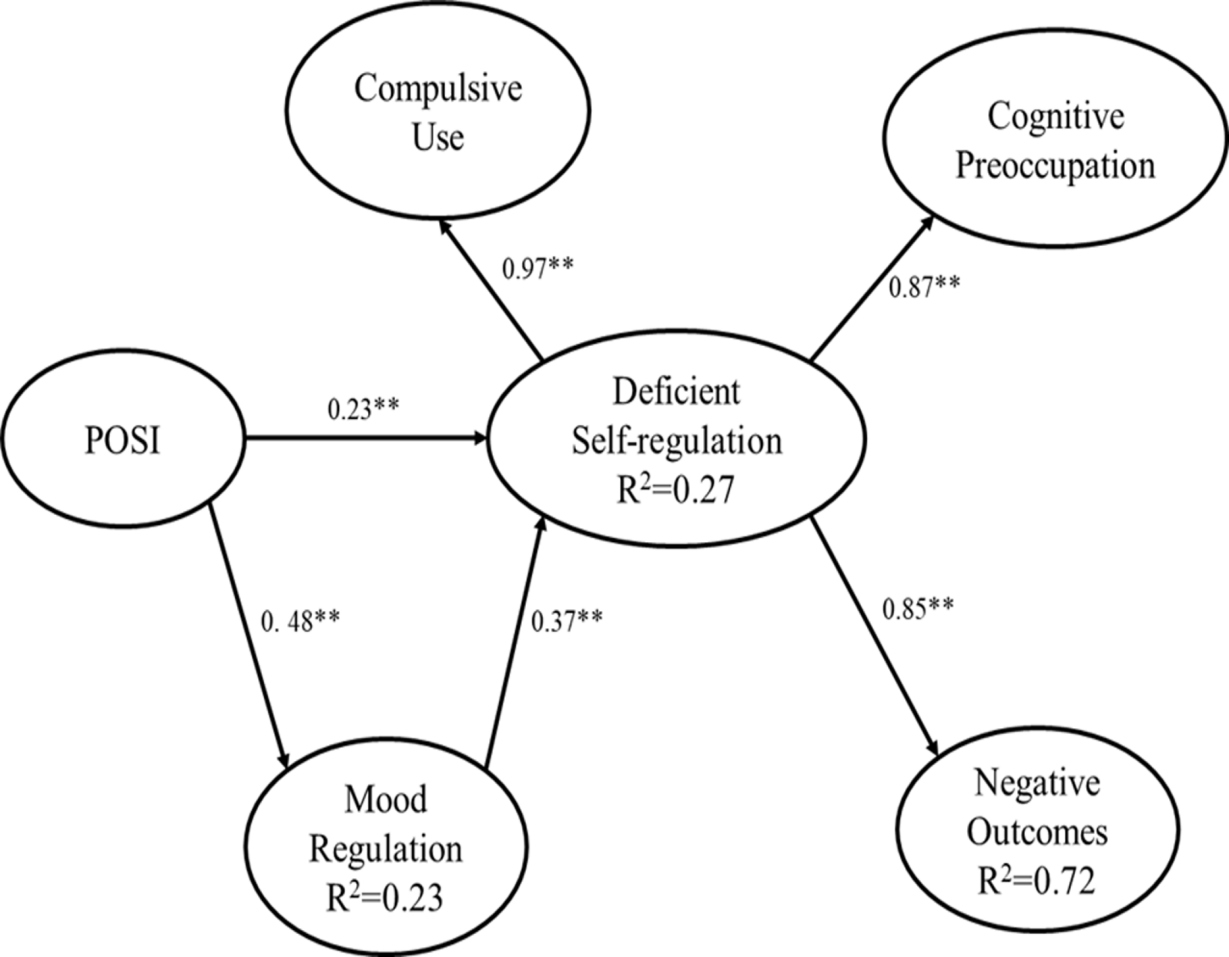
Standardized coefficient estimates of structural equation model of Problematic Social Media Use Scale. POSI stands for preference for online social interaction, ** stands for *p* < .001.

The factorial structure including both the five first-order factors and the second-order factor provided a good fit to the data. Thus, Caplan’s original model was tested. The second-order factor model showed a good fit of the data (SRMR = 0.04, RMSEA = 0.066, 90% CI [0.060–0.073]). The direct and indirect effects are shown in Fig. 2.

## Discussion

The present study validated a Chinese adaptation of the GPIUS2 for social-media use (PSMUS) and suggested Caplan’s cognitive-behavioral model (Caplan, 2010) as appropriate in the context of PUSM among Chinese undergraduates. The five first-order factors exhibited robust reliability, validity, and measurement invariance across sexes, with SEM fully supporting the hypothesized structure.

Of note, EFA supported the five-factor model but item 12, which loaded onto the “compulsive internet use” factor in the original version, exhibited high factor loadings on both “compulsive use” and “POSI” in our study. In another Japanese study, item 12 loaded onto a “cognitive preoccupation” factor, and this difference may be attributed to cultural differences related to obsessive-compulsive symptoms (Cervin et al., 2022; Højgaard et al., 2017; Matsunaga et al., 2008; Yoshimura et al., 2022). Obsessive thoughts about social media may be more likely to be perceived as a loss of behavioral control (a core feature of compulsive use) rather than a preference for online interaction in Chinese cultural contexts. The findings from this study thus enhances the model’s cultural validity for Chinese populations, and such differences suggest a need to consider cultural contexts when adapting Western-developed scales to Eastern population (Caplan, 2003; Yoshimura et al., 2022). Thus, item 12 was loaded onto “compulsive use.” CFA subsequently showed a good fit for both the five first-order factors and the four second-order factors models which aligns with some previous studies (Assunção & Matos, 2017; Laconi et al., 2018; Marino et al., 2017; Moretta & Buodo, 2018; Yoshimura et al., 2022). Different from other findings that have suggested insufficient construct validity of the model (Laconi et al., 2018) or have needed item 12 to be loaded onto the cognitive preoccupation factor for a better fit (Yoshimura et al., 2022), this Chinese version of the PSMUS showed a better fit and stability. Such differences may be attributed to variations in cultural norms and patterns of usage of social media. Previous researchers have asserted that such differences highlight an interaction between obsessive thoughts and compulsive use of social media, thus reflecting distinctive manifestations of reduced self-regulation (Fioravanti, Primi & Casale, 2013). Future studies including samples from different parts of the world should test the possible role of cultural differences in determining distinctive manifestations of poor self-regulation of usage of social media.

In alignment with previous findings (Assunção & Matos, 2017; Laconi et al., 2018; Yoshimura et al., 2022), the SEM supported the hypothesized structural relationships among model constructs. Specifically, the results highlighted that POSI is directly associated with mood regulation. Furthermore, both POSI and mood regulation are directly related to deficient self-regulation, which, in turn, is associated with negative outcomes. Moreover, POSI is indirectly associated with deficient self-regulation through mood regulation. Deficient self-regulation indirectly mediates the relationship between POSI and negative outcomes as well as between mood regulation and negative outcomes. Moreover, our previous study investigating the relationship between PUSM and psychological distress also found that more severe PUSM was associated with higher daily active and passive social media engagement, as well as elevated coping, conformity, enhancement motives, and stress symptoms (Li et al., 2025). Given the strength of path coefficients from the SEM results, some targeted interventions, such as implementing emotion-regulation training courses, developing offline social skill workshops, and offering time management guidance for social media use, are suggested. Such interventions may reduce students’ reliance on social media for mood adjustment, build confidence in face-to-face interactions, enhance self-regulation capacity, and mitigate the negative impacts of PUSM on academic performance and mental health (LaMontagne et al., 2024).

On the whole, the combined effect of POSI, mood regulation, and deficient self-regulation resulted in negative outcomes of PUSM, explaining 72% of the variance, which is higher than that in Japanese adults (Yoshimura et al., 2022), but lower than in Portuguese adolescents (Assunção & Matos, 2017) and an Italian sample (Moretta & Buodo, 2018). Differences in the target population and cultural norms may be contributing factors. The Portuguese sample was comprised of adolescents aged between 14 and 18 years, whereas the Japanese study recruited adults with an interquartile age range of 22–43 years. Participants in this study filled the age gap by using the same scale in different regions. Some researchers have proposed that the flow experience during use of social media may be a significant risk factor in the development of PUSM (Brailovskaia, Schillack & Margraf, 2020). Adolescents exhibit higher novelty-seeking (Bottaro & Faraci, 2022), difficulties in maintaining offline relationships (Waytz & Gray, 2018), and poor self-control (Spada, 2014). These characteristics may contribute to relationships with online social environments, which may address needs for social engagement. In contrast, older adults tend to exhibit higher self-regulation capacities. POSI and using social media for mood regulation warrant particular attention (Svicher, Fioravanti & Casale, 2021) and should be further studied in relation to both age and cultural differences in order to promote personalized prevention programs and treatment of PUSM.

From a clinical perspective, the present study provides a reliable and theory-driven tool that can be used to assess PUSM in Chinese college students. The findings suggest an important role for mood regulation and deficient self-regulation. Accordingly, interventions to reduce PUSM could prioritize enhancing skills to improve emotion regulation and foster adaptive coping strategies, as these factors were more related to negative outcomes than a preference for online social interaction. Speculatively, this implies that university-based initiatives could integrate modules on emotional awareness, mindfulness, and stress management into broader student wellness frameworks.

The present findings should be interpreted considering methodological limitations. First, although this is the first study evaluating the cognitive-behavioral model of PUSM in Chinese college students, its cross-sectional nature precludes determination of cause–effect relationships between variables, including regarding the temporal order of key factors such as POSI, mood regulation, and negative outcomes. Therefore, longitudinal studies involving Chinese college students are needed in order to better test the stability of the scale and examine potential causal relations between the constructs. Second, the use of a convenient sample, self-report data, and undifferentiated types of social media platforms (*e.g.*, short-video apps *vs.* messaging apps), which may include insufficient representation across diverse age groups, patterns or distinct levels of social media use (including with respect to self-selection bias related to the recruitment strategy and other factors), may limit the generalizability of the findings. Additionally, the present participants resided in urban areas, which also limited the generalizability to all Chinese college students. While we aimed for a large sample and implemented electronic screening and rigorous manual review procedures, we cannot exclude sampling biases, including with respect to over-representation of people with heavy use of social media. As such, we suggest caution when drawing conclusions from these data. Third, the lack of direct assessment of co-occurring psychopathologies limits the study of the relationship between PUSM dimensions and psychological distress. Future studies should improve sampling methods, include objective measures, design samples with more balanced coverage of age groups, social media types, and social media use levels, assess co-occurring psychopathologies, and collect information from other sources (*e.g.*, families or friends) to investigate further the proposed theoretical model and generalizability of the findings and to reduce potential biases.

## Conclusions

Overall, the PSMUS showed good psychometric properties in Chinese college students. The strong association between mood regulation and deficient self-regulation suggests the potential value of focusing on improving emotion-regulation difficulties in interventions targeting PUSM.

##  Supplemental Information

10.7717/peerj.21138/supp-1 Supplemental Information 1 Raw data

10.7717/peerj.21138/supp-2 Supplemental Information 2 Items of Problematic Social Media Use Scale

10.7717/peerj.21138/supp-3 Supplemental Information 3 STROBE checklist

10.7717/peerj.21138/supp-4 Supplemental Information 4 Codebook

## Additional Information and Declarations

### Data Availability

The following information was supplied regarding data availability: The raw data is available in the Supplemental File.

## References

[ref-1] Anish KR, Abraham KS, Jose J, Francis PNM, Joseph PA (2024). Navigating the web of influence: a bibliometric analysis of social media addiction. Cureus.

[ref-2] Assunção RS, Matos PM (2017). The generalized problematic internet use scale 2: validation and test of the model to Facebook use. Journal of Adolescence.

[ref-3] Bergaoui E, Bouallagui A, Hkiri A, Zrelli M, Moalla M, Amri G, Ghachem R (2024). Social media addiction among college students in Tunisia. L’Encephale.

[ref-4] Boer M, Van den Eijnden RJJM, Boniel-Nissim M, Wong S-L, Inchley JC, Badura P, Craig WM, Gobina I, Kleszczewska D, Klanšček HJ, Stevens GWJM (2020). Adolescents’ intense and problematic social media use and their well-being in 29 countries. Journal of Adolescent Health, Understanding Adolescent Health and Wellbeing in Context: Cross-National Findings from the Health Behaviour in School-Aged Children Study.

[ref-5] Bottaro R, Faraci P (2022). The use of social networking sites and its impact on adolescents’ emotional well-being: a scoping review. Current Addiction Reports.

[ref-6] Brailovskaia J, Margraf J (2017). Facebook addiction disorder (FAD) among German students—a longitudinal approach. PLOS ONE.

[ref-7] Brailovskaia J, Schillack H, Margraf J (2020). Tell me why are you using social media (SM)! Relationship between reasons for use of SM, SM flow, daily stress, depression, anxiety, and addictive SM use—an exploratory investigation of young adults in Germany. Computers in Human Behavior.

[ref-8] Brand M, Antons S, Bőthe B, Demetrovics Z, Fineberg NA, Jimenez-Murcia S, King DL, Mestre-Bach G, Moretta T, Müller A, Wegmann E, Potenza MN (2025). Current advances in behavioral addictions: from fundamental research to clinical practice. American Journal of Psychiatry.

[ref-9] Brand M, Rumpf H-J, Demetrovics Z, Müller A, Stark R, King DL, Goudriaan AE, Mann K, Trotzke P, Fineberg NA, Chamberlain SR, Kraus SW, Wegmann E, Billieux J, Potenza MN (2020). Which conditions should be considered as disorders in the International Classification of Diseases (ICD-11) designation of other specified disorders due to addictive behaviors?. Journal of Behavioral Addictions.

[ref-10] Caplan SE (2003). Preference for online social interaction: a theory of problematic internet use and psychosocial well-being. Communication Research.

[ref-11] Caplan SE (2010). Theory and measurement of generalized problematic internet use: a two-step approach. Computers in Human Behavior, Advancing Educational Research on Computer-Supported Collaborative Learning (CSCL) Through the Use of GStudy CSCL Tools.

[ref-12] Casale S, Fioravanti G (2018). Why narcissists are at risk for developing Facebook addiction: the need to be admired and the need to belong. Addictive Behaviors.

[ref-13] Cervin M, Miguel EC, Güler AS, Ferrão YA, Erdoğdu AB, Lazaro L, Gökçe S, Geller DA, Yulaf Y, Başgül ŞS, Özcan Ö, Karabekiroğlu K, Fontenelle LF, Yazgan Y, Storch EA, Leckman JF, do Rosário MC, Mataix-Cols D (2022). Towards a definitive symptom structure of obsessive-compulsive disorder: a factor and network analysis of 87 distinct symptoms in 1,366 individuals. Psychological Medicine.

[ref-14] Cheng C, Lau Y, Chan L, Luk JW (2021). Prevalence of social media addiction across 32 nations: meta-analysis with subgroup analysis of classification schemes and cultural values. Addictive Behaviors.

[ref-15] China Internet Network Information Center (2025). The 53rd Statistical Report on Internet Development in China. https://www3.cnnic.cn/n4/2024/0322/c88-10964.html.

[ref-16] Davis RA (2001). A cognitive-behavioral model of pathological Internet use. Computers in Human Behavior.

[ref-17] Dell’Osso B, Di Bernardo I, Chamberlain S, Fineberg N (2021). Learning to deal with problematic usage of the internet/revised edition. European Cooperation in Science and Technology.

[ref-18] Fioravanti G, Primi C, Casale S (2013). Psychometric evaluation of the generalized problematic internet use scale 2 in an Italian sample. Cyberpsychology, Behavior and Social Networking.

[ref-19] Gopakumar G, Surathkumaar H, Ramkumar T, Aljin V, Viswanath S, Joseph J (2025). Prevalence of social media addiction and its determinants among college students in Chengalpattu District, Tamil nadu. Cureus.

[ref-20] Haddad JM, Macenski C, Mosier-Mills A, Hibara A, Kester K, Schneider M, Conrad RC, Liu CH (2021). The impact of social media on college mental health during the COVID-19 pandemic: a multinational review of the existing literature. Current Psychiatry Reports.

[ref-21] Halladay J, Freibott CE, Lipson SK, Zhou S, Eisenberg D (2024). Trends in the co-occurrence of substance use and mental health symptomatology in a national sample of US post-secondary students from 2009 to 2019. Journal of American College Health.

[ref-22] Højgaard DRMA, Mortensen EL, Ivarsson T, Hybel K, Skarphedinsson G, Nissen JB, Valderhaug R, Dahl K, Weidle B, Torp NC, Grados M, Lewin AB, Melin KH, Storch EA, Wolters LH, Murphy TK, Sonuga-Barke EJS, Thomsen PH (2017). Structure and clinical correlates of obsessive—compulsive symptoms in a large sample of children and adolescents: a factor analytic study across five nations. European Child & Adolescent Psychiatry.

[ref-23] Hosen MJ, Eva SA, Rahman MM, Ibrahim Md, Lira UF, Hossain AB, Shill MC, Uddin MdJ (2021). Health impacts of excessive use of Facebook among university students in Bangladesh. Heliyon.

[ref-24] Hou X-L, Wang H-Z, Hu T-Q, Gentile DA, Gaskin J, Wang J-L (2019). The relationship between perceived stress and problematic social networking site use among Chinese college students. Journal of Behavioral Addictions.

[ref-25] Hussain Z, Griffiths MD (2021). The Associations between problematic social networking site use and sleep quality, attention-deficit hyperactivity disorder, depression, anxiety and stress. International Journal of Mental Health and Addiction.

[ref-26] Kim J, LaRose R, Peng W (2009). Loneliness as the cause and the effect of problematic internet use: the relationship between internet use and psychological well-being. CyberPsychology & Behavior.

[ref-27] Kolhar M, Kazi RNA, Alameen A (2021). Effect of social media use on learning, social interactions, and sleep duration among university students. Saudi Journal of Biological Sciences.

[ref-28] Kuss DJ, Griffiths MD (2017). Social networking sites and addiction: ten lessons learned. International Journal of Environmental Research and Public Health.

[ref-29] Laconi S, Kaliszewska-Czeremska K, Tricard N, Chabrol H, Kuss DJ (2018). The generalized problematic internet use scale-2 in a French sample: psychometric evaluation of the theoretical model. L’Encéphale.

[ref-30] LaMontagne LG, Doty JL, Diehl DC, Nesbit TS, Gage NA, Kumbkarni N, Leon SP (2024). Acceptability, usage, and efficacy of mindfulness apps for college student mental health: a systematic review and meta-analysis of RCTs. Journal of Affective Disorders.

[ref-31] LaRose R, Lin CA, Eastin MS (2003). Unregulated internet usage: addiction, habit, or deficient self-regulation?. Media Psychology.

[ref-32] Lee M-S, Potenza MN, Bhang S-Y (2025). Applying the interaction of person-affect-cognition-execution model to addictive behaviors in east asian countries: feasibility and considerations. Journal of the Korean Academy of Child and Adolescent Psychiatry.

[ref-33] Li M, Hao Y, Wang Y, Morris R, Wang X, Liu T, Chen S, Moretta T (2025). Prevalence, motivation, psychological distress, and gender differences of problematic social media use among chinese college students. Journal of Psychiatric Research.

[ref-34] Luo W, Zhong B-L, Chiu HF-K (2021). Prevalence of depressive symptoms among chinese university students amid the COVID-19 pandemic: a systematic review and meta-analysis. Epidemiology and Psychiatric Sciences.

[ref-35] Marino C, Vieno A, Altoè G, Spada MM (2017). Factorial validity of the problematic Facebook use scale for adolescents and young adults. Journal of Behavioral Addictions.

[ref-36] Matsunaga H, Maebayashi K, Hayashida K, Okino K, Matsui T, Iketani T, Kiriike N, Stein DJ (2008). Symptom structure in Japanese patients with obsessive-compulsive disorder. The American Journal of Psychiatry.

[ref-37] Moretta T, Buodo G (2018). Modeling problematic Facebook Use: highlighting the role of mood regulation and preference for online social interaction. Addictive Behaviors.

[ref-38] Moretta T, Buodo G, Demetrovics Z, Potenza MN (2022). Tracing 20 years of research on problematic use of the internet and social media: theoretical models, assessment tools, and an agenda for future work. Comprehensive Psychiatry.

[ref-39] Musetti A, Grazia V, Alessandra A, Franceschini C, Corsano P, Marino C (2022). Vulnerable narcissism and problematic social networking sites use: focusing the lens on specific motivations for social networking sites use. Healthcare.

[ref-40] Office of the Surgeon General (OSG) (2023). The US Surgeon General’s Advisory. https://www.hhs.gov/sites/default/files/sg-youth-mental-health-social-media-advisory.pdf.

[ref-41] Pirdehghan A, Khezmeh E, Panahi S (2021). Social media use and sleep disturbance among adolescents: a cross-sectional study. Iranian Journal of Psychiatry.

[ref-42] Sampasa-Kanyinga H, Lewis RF (2015). Frequent use of social networking sites is associated with poor psychological functioning among children and adolescents. Cyberpsychology, Behavior, and Social Networking.

[ref-43] Schermelleh-Engel K, Moosbrugger H, Müller H (2003). Evaluating the fit of structural equation models: tests of significance and descriptive goodness-of-fit measures. Methods of Psychological Research Online.

[ref-44] Seehuus M, Burt KB, Moeller RW (2025). College student mental health is stable across college career, but declining over time. Journal of Adolescence.

[ref-45] Soraci P, Demetrovics Z, Bevan N, Pisanti R, Servidio R, Di Bernardo C, Chini E, Griffiths MD (2025). FoMO and psychological distress mediate the relationship between life satisfaction, problematic smartphone use, and problematic social media use. International Journal of Mental Health and Addiction.

[ref-46] Spada MM (2014). An overview of problematic internet use. Addictive Behaviors.

[ref-47] Statista (2025). Number of social media users worldwide from 2017 to 2028. https://www.statista.com/statistics/278414/number-of-worldwide-social-network-users/.

[ref-48] Su B, Xiao X, Cheng Y, Liu C, Yang C (2025). Trajectories of depressive symptom among college students in China during the COVID-19 pandemic: association with suicidal ideation and insomnia symptoms. Suicide and Life-Threatening Behavior.

[ref-49] Svicher A, Fioravanti G, Casale S (2021). Identifying the central symptoms of problematic social networking sites use through network analysis. Journal of Behavioral Addictions.

[ref-50] Thomas J, Verlinden M, Beyahi FA, Bassam BA, Aljedawi Y (2022). Socio-demographic and attitudinal correlates of problematic social media use: analysis of Ithra’s 30-nation digital wellbeing survey. Frontiers in Psychiatry.

[ref-51] Violato C, Hecker KG (2007). How to use structural equation modeling in medical education research: a brief guide. Teaching and Learning in Medicine.

[ref-52] Waytz A, Gray K (2018). Does online technology make us more or less sociable? A preliminary review and call for research. Perspectives on Psychological Science.

[ref-53] Wu W, Huang L, Yang F (2024). Social anxiety and problematic social media use: a systematic review and meta-analysis. Addictive Behaviors.

[ref-54] Xie J-Q, Rost DH, Wang F-X, Wang J-L, Monk RL (2021). The association between excessive social media use and distraction: an eye movement tracking study. Information & Management.

[ref-55] Yoshimura S, Shibata M, Kyuragi Y, Kobayashi K, Aki M, Murai T, Fujiwara H (2022). The Japanese version of the generalized problematic internet use scale 2 (GPIUS2): psychometric evaluation and analysis of the theoretical model. PLOS ONE.

[ref-56] Zhang J, Marci T, Marino C, Canale N, Vieno A, Wang J, Chen X (2024). Factorial validity of the problematic social media use scale among Chinese adults. Addictive Behaviors.

[ref-57] Zhong B-L, Chen S-L, Tu X, Conwell Y (2017). Loneliness and cognitive function in older adults: Findings from the chinese longitudinal healthy longevity survey. Journals of Gerontology. Series B, Psychological Sciences and Social Sciences.

