# Supplementary material for: Reliability, factor structure, and criterion validity: testing the problematic social media use scale in Chinese college students
Source: PeerJ. 2026 May 11;14:e21138. doi: 10.7717/peerj.21138 (PMC13175062; doi:10.7717/peerj.21138)
Supplement: Supplemental Information 2 [file peerj-14-21138-s002.docx]

Items of Problematic Social Media Use Scale:

1. I prefer networking over face-to-face communication.

2. Online social interaction is more comfortable for me than face-to-face interaction.

3. I prefer to communicate with people online rather than in person.

4. I prefer to communicate with others online when I was feeling isolated.

5. I have used the networking to make myself feel better when I was down.

6. I have used the social media to make myself feel better when I was upset.

7. When I haven’t been online for some time, I become preoccupied with the thought of going online.

8. I would feel lost if I was unable to use social media.

9. I think obsessively about logging in social media when I am offline.

10. I find it difficult to control the time that I spend on social media.

11. I find it difficult to control my social media use.

12. When offline, I have a hard time trying to resist the urge to go social media.

13. My social media use has made it difficult for me to manage my life.

14. I have missed social engagements or activities because of my social media use.

15. My social media use has created problems for me in my life.
