# Supplementary material for: Reliability, factor structure, and criterion validity: testing the problematic social media use scale in Chinese college students
Source: PeerJ. 2026 May 11;14:e21138. doi: 10.7717/peerj.21138 (PMC13175062; doi:10.7717/peerj.21138)
Supplement: Supplemental Information 3 [file peerj-14-21138-s003.docx]

**STROBE Checklist**

**Title, Abstract, and Introduction**

1. **Title**: Include the study design (e.g., cross-sectional study) - **Abstract** and **Introduction** sections.
2. **Abstract**: Provide a structured summary of the study (background, methods, results, conclusions) - **Abstract** section (lines 1-10).
3. **Introduction**: Explain the scientific background and rationale for the study - **Introduction** section (lines 11-30).

**Methods**

1. **Study Design**: Describe the study design (cross-sectional) - **Materials & Methods** section (lines 65-66).
2. **Setting**: Describe the setting, locations, and relevant dates - **Materials & Methods** section (lines 67-69).
3. **Participants**: Describe the eligibility criteria and recruitment methods - **Materials & Methods** section (lines 70-75).
   - Inclusion criteria: using social media, being a college student, being 18 years or older.
   - Exclusion criteria: having identical answer options for all questions, finishing the survey in less than 120 seconds.
4. **Variables**: Clearly define all variables and measurement tools - **Materials & Methods** section (lines 76-80).
   - Main variable: Problematic Social Media Use Scale (PSMUS).
5. **Data Collection**: Describe the methods of data collection - **Materials & Methods** section (lines 81-83).
   - Data collected via QR code and website link delivery through WeChat, QQ Zone, and Douban.
6. **Bias**: Discuss potential biases and how they were minimized - **Discussion** section (lines 360-365).
   - Limitations include cross-sectional design, convenience sampling, and self-report data.
7. **Sample Size**: Explain the sample size calculation - **Materials & Methods** section (lines 84-85).
   - Sample size met the recommended size for Structural Equation Modeling (SEM).
8. **Statistical Methods**: Describe the statistical methods used - **Materials & Methods** section (lines 86-95).
   - Methods include exploratory factor analysis (EFA), confirmatory factor analysis (CFA), and structural equation modeling (SEM).

**Results**

1. **Participants**: Report the number of participants and response rate - **Results** section (lines 100-102).
   - Total of 788 college students participated.
2. **Descriptive Data**: Provide descriptive data for the sample - **Results** section (lines 103-105).
   - Sociodemographic characteristics reported in Table 1.
3. **Outcome Data**: Report the main outcome data - **Results** section (lines 106-120).
   - EFA and CFA results, including factor loadings and model fit indices (lines 106-115).
   - SEM results, including direct and indirect effects (lines 116-120).
4. **Other Analyses**: Report any other analyses performed - **Results** section (lines 121-125).
   - Measurement invariance analysis across sexes.

**Discussion**

1. **Key Results**: Summarize key results and compare with previous studies - **Discussion** section (lines 130-140).
   - Results support the psychometric properties of the PSMUS in Chinese college students.
2. **Interpretation**: Discuss the interpretation of the results - **Discussion** section (lines 141-150).
   - POSI and mood regulation are significant predictors of deficient self-regulation and negative outcomes.
3. **Limitations**: Discuss the limitations of the study - **Discussion** section (lines 151-155).
   - Cross-sectional design, convenience sampling, lack of psychopathology measures.
4. **Generalizability**: Discuss the generalizability of the findings - **Discussion** section (lines 156-160).
   - Results may be applicable to other Eastern cultures but need further validation.
5. **Implications**: Discuss the implications for future research and practice - **Discussion** section (lines 161-165).
   - Suggests interventions targeting emotion regulation and coping strategies.

**Other Information**

1. **Ethics Approval**: Report ethics approval and consent - **Materials & Methods** section (lines 62-64).
   - Approved by the Institutional Review Board of the Second Xiangya Hospital of Central South University.
2. **Funding**: Report funding sources and conflicts of interest - **Acknowledgements** section (lines 370-375).
