# Supplementary material for: Reliability, factor structure, and criterion validity: testing the problematic social media use scale in Chinese college students
Source: PeerJ. 2026 May 11;14:e21138. doi: 10.7717/peerj.21138 (PMC13175062; doi:10.7717/peerj.21138)
Supplement: Supplemental Information 4 [file peerj-14-21138-s004.docx]

Codebook

Gender:

1 – male

2 – female

Item 1-15:

1 – Strongly disagree
2 – Highly disagree
3 – Moderately disagree
4 – Disagree
5 – Moderately agree
6 – Agree
7 – Highly agree
8 – Strongly agree
